# Prevalence of antibodies to *Brucella* species in commercial raw bovine milk in Southwestern Uganda

**DOI:** 10.1186/s13104-017-2537-5

**Published:** 2017-09-08

**Authors:** Monicah Kamwine, Patrick Orikiriza, Kabanda Taseera, Jacob Stanley Iramiot, Patrick Ojuka, Steven Ikiriza, Jeninah Atwebembeire, Duncan Otieno, Silver Tweshengyereze, Juliet Mwanga-Amumpaire, Joel Bazira, Yap Boum

**Affiliations:** 1 Department of Microbiology, Mbarara University of Science and Technology, P.O. Box 1410, Mbarara, Uganda; 2 Epicentre Mbarara Research Centre, P. O Box 1956, Mbarara, Uganda; 3 Department of Microbiology, Busitema University, Busitema, Uganda; 4 Dairy Development Authority, Kampala, Uganda; 5 GBK Group of Companies, Mbarara, Uganda

**Keywords:** Brucellosis, Diagnosis, Indirect ELISA, Milk ring test

## Abstract

**Objectives:**

The purpose and objective of this research was to explore the prevalence of antibodies against *Brucella* species in raw milk samples collected in Southwestern Uganda, one of the biggest milk producing regions in the Country. We hypothesized that there is a high level of antibodies in milk samples from this region. This builds more evidence to other studies in the region on the level contamination of raw milk.

**Results:**

A total of 185 raw milk samples, collected from dairy farms and factories in southwestern region, were tested for antibodies to *Brucella* spp. using the milk ring test (MRT) and indirect Enzyme-Linked Immunosorbent Assay (i-ELISA).We found a prevalence of 26.5% (49/185) by the two methods. This is related to previous reports in the region and adds more evidence on the need for further investigations to confirm the source of these antibodies and their relationship with disease in milk producing animals.

## Background

Brucellosis is a common disease in many cattle keeping countries communities consuming raw milk [1]. The disease is mainly zoonotically transmitted by species of *Brucella melitensis*, *Brucella abortus*, *Brucella suis*, among others [2]. Although definitive isolation is the gold standard, it is time-consuming with low-sensitivity and requires biosafety level-3 making serological tests favorable in low resource settings [3].

The MRT is routinely used as a screening test on fresh but not pasteurized milk [4]. Despite its wide use, it is prone to false positive reactions especially in milk containing colostrum and at the end of lactation in cows with a hormonal disorder or mastitis [5]. Other screening tests like i-ELISA are recommended along side MRT inorder to confirm presence of antibodies [6].

In Uganda, there is a high presence of cattle. The national demographic and health survey estimates that 14.5 and 23.2% of the urban and rural populations own local cattle [7]. It is also estimated that there is a total herd population of 14 million [8] producing about 1.9 billion liters of milk per year [9]. About 30% of this milk is consumed on the farm and may raise concern about zoonotic infections. The annual per capita consumption is estimated at 58 litres/person/annum [9]. Beyond East Africa, Uganda exported 6555 and 10,803 millions tons of milk in 2009 and 2011. In addition, 14,187 million tons of milk products were exported in 2011; 60% of which were ultra heat treated [9, 10].

A recent study on farms in Southwestern Uganda estimated the prevalence of brucella antibodies at 29% using MRT alone [13]. There is limited data on the prevalence beyond the farm. In this study, we aimed to determine the prevalence of Brucella antibodies in southwestern region in order to guide the national disease control program.

## Methods

### Study location

The study was carried out in greater Mbarara region, which is located in Southwestern Uganda.

The equatorial temperate climate includes two rainy seasons (March–May and September–December) with mean minimum temperature of 14.6 °C and maximum annual temperature of 30.8 °C. The climate and annual rainfall of 822 mm occur in 114 rainy days in the year and is favorable for dairy production [11].

### Definition of dairies

We considered only dairies where farmers were able to accumulate more than 500 l of milk per day.

### Sample collections and handling

Between August and September 2014 we performed a descriptive cross sectional serosurvey. A multi-stage sampling technique based on districts was adopted. We started by generating a list of possible farms and factories producing milk at the district. This was compiled with the assistance of District Dairy Development Authority (DDA) officials. The list comprised of dairies from four districts: Kiruhura, Mbarara, Bushenyi and Isingiro. These four were purposively selected because they are the main dairy producers according to DDA. In Kiruhura, Mbarara, Bushenyi and Isingiro, 96, 59, 18, and 12 dairies respectively were conveniently selected, taking into account their spatial distribution (Table 1). We started sampling from the diaries at the district and ended at the local council level. In all the cases, the districts had bigger collection points compared to the local council. From each dairy the owners were asked to consent to be part of the study. The consent process was done in private to avoid interference and also ensure confidentiality. Consenting participants were explained about the study and were given a right to withdraw at anytime if they felt uncomfortable. The consent form was translated in the local language (Runyankole). After consenting, we collected 10 ml of raw milk from randomly selected coolers and kept all samples refrigerated at 4 °C prior to laboratory investigations. These included a mixture of fresh to 2 days’ old milk collected samples.

**Table 1:** Prevalence of antibodies against *Brucella* spp. in raw milk samples by districts

District	No. of samples examined	No. of positive samples	Prevalence (%)	95% CI
Kiruhura	96	39	40.6	30.7–51.1
Mbarara	59	33	55.9	42.4–66.8
Bushenyi	18	10	55.6	51.6–97.9
Isingiro	12	8	66.7	44.4–97.4
Total	185	90	48.6	41.2–56.1

### Laboratory methods

We processed the samples at Epicentre Mbarara Research Centre and Milk Research Laboratory, Mbarara University of Science and Technology. All specimens were tested with two methods: (1) the milk ring test (MRT) and (2) Indirect Enzyme-linked immune absorbent assays (i-ELISA). We performed the MRT as described previously [6]. In brief we added 30 μl of *B. abortus* Bang Ring Antigen (State Biological Laboratory, Institute of Veterinary Preventive Medicine, Ranipet, India) to 1 ml of raw milk that had been kept at 4 °C for 24 h at the Milk Research Laboratory. Samples were incubated for 1 h at 37 °C together with positive and negative controls. Agglutinated Brucella cells were picked up by fat globules as they rose, forming a dark cream layer on top of the sample as previously described [6]. The reading was done and a positive reaction was indicated by a purple layer over a white column of milk [3]. A negative test was indicated when the color of the underlying milk was more blue than that of the cream layer.

I-ELISA was performed with the ID Screen^®^ Brucellosis milk indirect assay (Innovative Diagnostics, Grabels, France). The assay detects antibodies against *Brucella* spp. in bovine, ovine and caprine milk. Detection of anti-Brucella antibodies was performed and interpreted according to manufacturer’s instructions [12].

### Statistical analysis

Data were entered into Microsoft Excel spreadsheets, and analyzed using Stata SE v12 software (College Station, Texas, USA). In this study, a serial testing protocol was used and therefore, a milk sample was considered positive for antibodies against *Brucella* spp. if it was positive for both the MRT and i-ELISA. We also compared the prevalence of antibodies against *Brucella* spp. between factories and farms, including individual milk coolers, milk bicycle carriers and the family’s farms, using Chi square test. Finally, we measured the degree of agreement between MRT and i-ELISA using Kappa statistics.

## Results

Between August and September 2014, we collected 185 raw milk samples from 185 dairies in Kiruhura, Mbarara, Bushenyi, and Isingiro districts of Southwestern Uganda. Among them, 51.9% (96/185) were collected from Kiruhura district, 31.9% (59/185) from Mbarara district, 9.7% (18/185) from Bushenyi district, and 6.5% (12/185) from Isingiro district (Table 1). Of these, 22 (11.9%) were from factories and 163 (88.1%) from farms (Table 2).

**Table 2:** Prevalence of antibodies against *Brucella* spp. in raw milk samples between factories and farms

Collection sites	Number of samples examined	Number of positive samples	Prevalence (%)	95% CI	Chi square, p value
Factories	22	18	81.82	59.7–94.8	0.004
Farms	163	72	44.17	36.4–52.1	

We found antibodies against *Brucella* spp. in 62 (33.5%) raw milk samples using the milk ring test alone and 90 (49.45%) with i-Elisa test (Table 3). However, the overall prevalence of antibodies against *Brucella* spp. was 26.5% using both methods, on the same samples (Table 1). We found the highest proportion of antibodies in Bushenyi 44.4%, [95% confidence interval (CI) 21.5–69.2] and the lowest in Kiruhura 14.6% (95% CI 8.2–23.2). However basing on the confidence intervals, the difference was not statistically significant among Kiruhura, Mbarara, Bushenyi and Isingiro (Table 1). We found a significantly higher prevalence in samples collected at factories than those collected directly from farms, using Chi square test (54.5% vs. 22.7%, p value = 0.004, Table 2). We also found agreement between MRT and i-ELISA methods (κ 0.40, p < 0.0001) (Table 4).

**Table 3:** Prevalence of antibodies against *Brucella* spp. in raw milk using i-ELISA and MRT

	Positive n (%)	Negative n (%)	Total	Chi square, p value
MRT	62 (33.5)	123 (66.5)	185	<0.001
i-ELISA	90 (49.5)	92 (50.5)	182^a^	
Total	152	215	367	

^a^ 3 invalids (doubtful)

**Table 4:** Comparison between MRT and i-ELISA in detection of antibodies against *Brucella* spp.

	i-ELISA			
MRT	Negative n (%)	Positive n (%)	Total	Chi square, p value
Negative (n)	80 (66.1)	41 (33.9)	121	<0.001
Positive (n)	12 (19.7)	49 (80.3)	61	
Total	92	90	182	

## Discussion

We detected antibodies to *Brucella* spp. at 62 (33.5%) and 90 (49.45%) using MRT and i-ELISA respectively in raw milk samples collected in the greatest milk producing areas of Southwestern Uganda. However, using a combination of the two screening methods, the antibodies were confirmed in 26.5% of samples. We considered a combination of two screening methods in order to increase accuracy of detection, considering that each of these has it’s limitations. Based on our findings, the antibody prevalence is generally comparable to what has been previously reported in this region. A recent study also found the level of antibodies at 29% in a rural part of Southwestern Uganda [13]. Our findings however differ from what was reported in central part of Uganda, with an equally high presence of cattle, where the level of antibodies was much lower at 1.2–3.3% but using i-ELISA alone [14]. This implies that there is relatively high prevalence of antibodies against *Brucella* spp. in raw milk in Southwestern Uganda. This is a potential risk considering that some cows could be harboring brucella pathogens and zoonotically infecting between themselves and humans.

Presence of antibodies in milk has been shown to project ongoing transmission. In one of the studies conducted in Uganda, presence of antibodies in human serum was strongly associated with a positive screening test on the milk samples from cattle of the farmer [13]. This therefore suggests that there is possibly a 26.5% chance of transmission. The population that consumes raw milk may be at increased risk of developing brucellosis [9]. Despite this assumption, further studies should look at the actual presence of brucella pathogens in the milk using molecular and cultural methods inorder to ascertain the extent of risk.

Although we didn’t find geographic differences we observed a difference among the collection sites. We observed a higher prevalence in milk collected from factories compared to farms. This could be explained by the fact that factories were purchasing the entire milk produced by selected farms and thus the high volumes could have contributed to this difference. Since the milk was collected at the factory before treatment, we believe that local farmers may have been biased by expected pasteurization process at the factory. Thus they were less likely to get concerned about the hygiene and conservation of milk before sending it. We also noted a higher percentage of antibodies in raw milk collected from Bushenyi although the proportion was not statistically significant.

Though we have not collected processed milk from these factories, we believe that the use of the pasteurization process, which is commonly performed at milk factories will reduce the risk of brucellosis in the population. There is evidence that pasteurization at a temperature of 62−63 °C applied 3 min is sufficient to destroy Brucella organisms [15].

Basing on single test, the prevalence of antibodies in milk would be overestimated. The MRT and i-ELISA estimated the rate at 62 (33.5%) and 90 (49.45%) respectively. Using a single test, high prevalence of antibodies in raw milk has been reported in some countries in East African region. In Tanzania, the rate was found at 56% in samples tested using MRT [16]. This figure could be an overestimation considering that only one method was used. Nevertheless, the high antibodies may be explained by the fact that about 90% of milk sales in Tanzania are in the hands of farmers who are known to harbour beliefs that milk is inherently hygienic [16]. However, in urban setting where milk is pasteurized and/or boiled before drinking, a lower prevalence of bovine brucellosis from milk has been reported. For example in Kampala, the capital city of Uganda, where antibody prevalence of 12.6% has been reported from marketed milk using the i-ELISA assay [17]. In contrast, in Kenya where standard pasteurization techniques are adopted [18], Kang’ethe et al. reported a proportion of 1% showing the impact of pasteurization on the antibody presence in raw milk samples [18].

We also found a significant difference in detection of antibodies against *Brucella* spp. between the MRT and i-ELISA assays; κ agreement of 0.40, p < 0.0001. While the sensitivity and specificity of MRT have been described at 85 and 95% respectively [19], the reported performance of i-ELISA for detection of antibodies against *Brucella* spp. in milk were much higher (sensitivity 98.5%, specificity 99.5%) [20, 21]. This highlights the importance of the two methods since a single test may under or over report the burden of the disease. Fortunately, presence of antibodies against *Brucella* spp. does not always reflect presence of Brucella pathogens. In one of the studies, ELISA detected antibodies in 21.4% of the milk samples, but only 7% was confirmed by PCR [22]. For that reason, confirmation of positive screening tests remains critical although most facilities continue to rely on single test results.

Nevertheless, the discrepancy encountered in this study using the two methods is comparable with studies performed in Sudan [19]. Therefore there is need for further studies to investigate field performance of MRT in low resources setting where there is high production and consumption of raw milk.

## Conclusion

We observed a high prevalence of antibodies against *Brucella* spp. in raw milk collected from dairies in Southwestern Uganda, the biggest producers of milk in the country. These findings need further confirmation by molecular and culture methods since presence of antibodies may not always indicate disease. This is important considering that raw milk consumption on the farm is a common practice in Uganda especially in rural settings. This will help public health experts to focus interventions appropriately.

## Limitations

- We were unable to confirm the discordant results between MRT and i-ELISA methods.
- We could not rule out possible contaminating organisms that may cause cross reactions, for example, bacteria causing mastitis in the milk samples.
- We were unable to confirm presence of pathogenic *Brucella* spp. in milk samples through culture or PCR based methods.
- The sample size from districts was small to make a generalizable conclusion.

## Abbreviations

ELISA: Enzyme Linked Immunosorbent Assay
MRT: milk ring test
WHO: World Health Organization
DDA: dairy drug authority
